# Diffusion Tensor Imaging in chronically shunted patients

**DOI:** 10.1186/2045-8118-12-S1-O31

**Published:** 2015-09-18

**Authors:** Kristy Tan, Adam L Sandler, Avital Meiri, Rick Abbott, James T Goodrich, Asif K Suri, Michael L Lipton, Mark E Wagshul

**Affiliations:** 1 Albert Einstein College of Medicine, USA; 2 Department of Neurological Surgery, Albert Einstein College of Medicine/Children's Hospital at Montefiore, Bronx, NY, USA

## Introduction

Chronically shunted patients with functioning shunts are often characterized as having slit or smaller than normal ventricles and chronic headaches. In this study, we used Diffusion Tensor Imaging (DTI) to quantitatively analyze the directionality of water diffusion in the internal capsule (IC) and the corpus callosum (CC) in these patients which have been shown to be affected in untreated hydrocephalus patients [1].

## Methods

Shunt-dependent patients who developed hydrocephalus as infants were selected. Preliminary results from 17 patients who suffer from chronic headaches (excluding patients with abnormally large ventricles) are shown in comparison to age and gender matched controls. DTI data was acquired using a single-shot EPI sequence with TE/TR=69/10000ms, voxel size= 2mm x 2mm x 2mm, max b-factor=800, num. of directions=32. White matter ROIs were manually drawn on high-resolution T1-weighted images with the aid of fractional anisotropy (FA) maps to locate the internal capsule (IC) (right and left, anterior and posterior limbs), genu and splenium of the corpus callosum (CC) and were verified by a neuroradiologist.

## Results

A statistically significant decrease (student t-test, p<0.05) in FA was found in the patient group compared to the controls in the right posterior (FA=0.61 vs 0.65, p=0.0045), left posterior (FA=0.61 vs 0.66, p<0.001) and left anterior IC (FA=0.56 vs 0.63, p=0.0031) as well as the splenium of the CC (FA=0.69 vs 0.80, p<0.001). No correlations were found between ventricular volumes with the DTI parameters.

## Conclusions

This study is the first to use DTI to study chronically shunted patients and shows evidence of white matter degradation based on decreases in FA in patients compared to controls. Previous studies have shown increased FA values in the IC and a decrease in the CC of patients and hypothesized that it was due to an increase of mechanical compression whilst others have hypothesized that a decrease in FA indicated secondary irreversible, degenerative changes [1-3]. These studies were performed on younger patients pre-treatment who usually have enlarged ventricles and we hypothesize that this may be the source of the difference coupled with the chronic shunt dependence within our patient group.
